# Effects of probiotic supplementation on subjective and objective sleep outcomes: an updated systematic review and meta-analysis of 39 randomized controlled trials

**DOI:** 10.3389/fpsyt.2026.1769331

**Published:** 2026-05-19

**Authors:** Keying Zhu, Jing Ouyang, Ping Liu, Xiaming Wang, Peng Kou, Jing Zhao

**Affiliations:** 1 Department of General Medicine, The Sixth Medical Center of People's Liberation Army (PLA) General Hospital, Beijing, China; 2 Department of Military Personnel Medical Affairs, The Sixth Medical Center of People's Liberation Army (PLA) General Hospital, Beijing, China

**Keywords:** meta-analysis, probiotics, PSQI, sleep, systematic review

## Abstract

**Introduction:**

Sleep disturbances are common and contribute to substantial functional and health-related burden. Probiotics have been proposed as a potential modulator of sleep via gut–brain axis mechanisms, yet the magnitude, consistency, and clinical significance of their effects remain uncertain. This study synthesized all randomized controlled trials evaluating the effect of probiotic supplementation on validated subjective and objective sleep outcomes, using harmonized change-score methodology and contemporary certainty-of-evidence grading.

**Methods:**

An updated search (March 31, 2022–October 1, 2025) was performed in PubMed, Scopus, and Web of Science to identify randomized placebo-controlled trials evaluating probiotics in adults or adolescents. Outcomes included global Pittsburgh Sleep Quality Index (PSQI) scores, PSQI subdomains, insomnia severity, objective sleep measures, and OSA Sleep Inventory MA (OSA-MA) factors. Effect sizes were synthesized as mean differences or standardized mean differences comparing change from baseline between probiotic and placebo groups. Random-effects models were used for all analyses.

**Results:**

Thirty-nine trials (n=4094 participants) were included. Probiotic supplementation significantly improved PSQI global scores (mean difference (MD) –0.71; 95% CI –1.23 to –0.20; very low certainty). Benefits were domain-specific, with significant improvements in daytime dysfunction (MD –0.08; 95% CI –0.11 to –0.05) and sleep time (MD 0.11; 95% CI 0.01 to 0.21), while other subdomains showed no consistent effects. Insomnia severity did not improve overall (SMD 0.17; 95% CI –1.15 to 1.49), although sensitivity analysis excluding an influential study indicated a significant reduction (standardized mean difference (SMD) –0.44; 95% CI –0.74 to –0.14; low certainty). Objective sleep outcomes showed modest improvements, including increased total sleep time (MD 13.82 minutes; 95% CI 1.15 to 26.49) and reduced awake time during sleep period (MD 5.15 minutes; 95% CI 0.43 to 9.87). Probiotics significantly improved OSA-MA Factor 1 (sleepiness on rising; MD 1.07; 95% CI 0.40 to 1.75) and Factor 4 (refreshing on rising; MD 1.18; 95% CI 0.42 to 1.94), with no significant differences in other factors.

**Conclusions:**

Probiotic supplementation is associated with small improvements in global subjective sleep quality and selected subjective sleep domains. However, effect sizes are modest, certainty of evidence is generally low, and the clinical meaningfulness of these findings remains uncertain.

**Systematic review registration:**

https://center6.umin.ac.jp/cgi-open-bin/ctr_e/ctr_view.cgi?recptno=R000057556, identifier UMIN000050539.

## 1 Introduction

Poor sleep and sleep disturbances are highly prevalent and confer substantial morbidity across physical, cognitive and psychiatric domains; even modest, persistent impairments in sleep quality or continuity are associated with impaired daytime function, reduced quality of life, and increased cardiometabolic and mental-health risk (1). To facilitate standardized assessment of subjective sleep quality in both clinical and research settings, several validated instruments have been developed. Among these, the Pittsburgh Sleep Quality Index (PSQI) is one of the most widely used tools for capturing global sleep quality and its component domains (2).

Interest in non-pharmacological, low-risk approaches to improve sleep has grown because of limitations of available therapies and the high prevalence of chronic sleep complaints. One area of active investigation is the microbiota–gut–brain axis, a bidirectional physiological network by which gut microbes can affect central nervous system function through neural (vagal and enteric), endocrine (including hypothalamic–pituitary–adrenal axis modulation), immune, and metabolic (microbial metabolite) pathways. This mechanistic framework provides biologically plausible routes through which live microbial interventions (probiotics) could influence sleep quality and related symptoms (3, 4).

Preclinical and human experimental work has identified several candidate mechanisms that may link specific probiotic strains to sleep modulation, including effects on tryptophan–serotonin metabolism, production or regulation of GABAergic signaling, attenuation of systemic and neuroinflammation, and normalization of HPA-axis hyperactivity (5). Randomized clinical trials testing defined probiotic strains (for example, *Lactobacillus gasseri*, *Lactobacillus plantarum*, and *Bifidobacterium breve* strains) have reported variable improvements in subjective sleep measures and, in some cases, objective sleep indices; mechanistic human data from trial cohorts further support HPA-axis modulation as one potential mediator (4, 6).

Several systematic reviews and meta-analyses have synthesized the early randomized evidence and reported modest improvements in global subjective sleep quality (most commonly PSQI) and in selected domain scores, while findings for objective sleep parameters and insomnia severity measures have been less consistent (7, 8). A recent comprehensive systematic review conducted by an independent research group summarized 15 randomized controlled trials and concluded that probiotics may improve PSQI scores and certain OSA-MA subscales (9), while emphasizing heterogeneity in populations, probiotic formulations, dosing, and outcome reporting.

Since the last published synthesis (9), the evidence base has expanded with additional randomized trials published after March 31, 2022, including studies reporting extractable pre–post change data and more detailed objective sleep outcomes (10–14). The present study represents an updated and independently conducted systematic review and meta-analysis that preserves the original clinical question and core eligibility framework, while incorporating newly available trials and applying consistent change-score analytic methods (ΔProbiotic − ΔPlacebo) across subjective and objective sleep outcomes. Given the growth in available randomized evidence and ongoing uncertainty regarding the magnitude and clinical relevance of probiotic effects on sleep, an updated synthesis was undertaken to provide a contemporary and comprehensive appraisal of the totality of evidence.

## 2 Materials and methods

### 2.1 Search strategy and selection criteria

This systematic review and meta-analysis was conducted in accordance with PRISMA guidelines (15). This study represents an updated systematic review and meta-analysis based on a previously published review with a prospectively registered protocol (UMIN000050539) (9). The previously published review was developed and conducted by an independent research team (9).

The current update was independently conducted while preserving the original clinical question, core eligibility criteria, outcome definitions, and focus on placebo-controlled randomized controlled trials in adults. All trials included in the prior review were retained, and the literature search was extended from the previous search date (March 31, 2022) to October 1, 2025. In accordance with PROSPERO guidance, a separate PROSPERO registration was not undertaken because the original protocol was publicly available and the present work extends that predefined methodological framework. A structured comparison of methodological features between the original review and the present update is provided in **Supplementary Table 1**.

The updated search was conducted through PubMed, Scopus, and Web of Science using controlled vocabulary and free-text terms related to probiotics and sleep outcomes. The full search strategy for each database is provided in the **Supplementary File**. No language, publication status, or geographical restrictions were applied at the search stage.

Randomized controlled trials evaluating probiotic supplementation compared with placebo in adults or adolescents were eligible if they reported extractable data for at least one validated subjective or objective sleep outcome. Trials using synbiotics, paraprobiotics, or multicomponent interventions in which the independent effect of probiotics could not be isolated were excluded. Cross-over studies were eligible if data were available for paired analysis or if baseline and post-intervention measurements could be extracted separately; however, none of the studies ultimately included in the meta-analysis employed a cross-over design.

The retrieved studies were then imported into Endnote V8 (Clarivate Analytics, Philadelphia, PA, USA) for duplicate screening and removal before the screening process which was carried out by two independent reviewers.

### 2.2 Data extraction and outcome measures

Two reviewers independently screened titles, abstracts, and full texts and extracted data using a standardized form. Extracted variables included study design, participant characteristics, probiotic strain(s), dose, formulation, treatment duration, and all sleep-related outcomes. When studies reported multiple time points, the end-of-intervention assessment was used to ensure comparability across trials. Discrepancies were resolved by consensus.

The primary outcome was change in global sleep quality measured by the Pittsburgh Sleep Quality Index (PSQI) (2). Secondary outcomes included PSQI subdomains, insomnia severity scales (Insomnia Severity Index [ISI] (16) or Athens Insomnia Scale [AIS] (17)), objective sleep parameters (e.g., total sleep time, REM-related efficiency, and minutes awake during the sleep period as reported in the original trials), and the five subdomains of the OSA Sleep Inventory MA version (OSA-MA) (18). For all outcomes, effect sizes were calculated as the difference in mean change from baseline between the probiotic and placebo groups, defined as: Δ = (Postprobiotic − Preprobiotic) − (Postplacebo − Preplacebo).

When change-score standard deviations were not reported, they were obtained using established formulas that reconstruct variance based on pre–post correlations, as recommended in the Cochrane Handbook for Systematic Reviews of Interventions (Chapter 6: Choosing effect measures and computing estimates of effect) (19). Evidence-based imputation methods were applied using correlation coefficients from studies reporting complete data or from validated assumptions in the sleep literature. For studies reporting medians and interquartile ranges, means and standard deviations were estimated using validated transformation methods when appropriate.

Because insomnia severity was measured using different validated instruments, standardized mean differences (Cohen’s d) were used for this outcome, while all other outcomes were analyzed using mean differences.

### 2.3 Risk of bias assessment

Risk of bias was assessed independently by two reviewers using the Cochrane Risk of Bias 2.0 tool (20), evaluating bias arising from the randomization process, deviations from intended interventions, missing outcome data, outcome measurement, and selective reporting. Any disagreements were adjudicated by a third reviewer.

### 2.4 Data synthesis and statistical analysis

Random-effects models using a restricted maximum likelihood (REML) estimator were used for all meta-analyses to account for anticipated clinical and methodological heterogeneity. Statistical heterogeneity was quantified using the I² statistic, where a value >50% indicated significant heterogeneity. Leave-one-out sensitivity analyses were performed for outcomes with significant heterogeneity or when influential studies were suspected. Small-study effects were evaluated using Egger’s regression when ≥10 studies were available for a given outcome; funnel plots were inspected visually when an outcome was presented by at least 10 trials.

Pre-specified subgroup analyses based on probiotic type, formulation, and dose intensity (≤1×10^6^ vs. ≥5×10^6^ CFU/day) could not be conducted due to insufficient reporting and lack of consistency across trials. Similarly, meta-regression was not feasible for these variables. All analyses were conducted using Stata V18 (StataCorp LLC, College Station, TX, USA).

### 2.5 Certainty of evidence

The certainty of evidence for each outcome was evaluated using the GRADE approach, considering risk of bias, inconsistency, indirectness, imprecision, and publication bias. Summary of Findings tables were generated to present effect estimates and certainty ratings across all major outcomes.

## 3 Results

### 3.1 Literature search results

**Figure 1:**
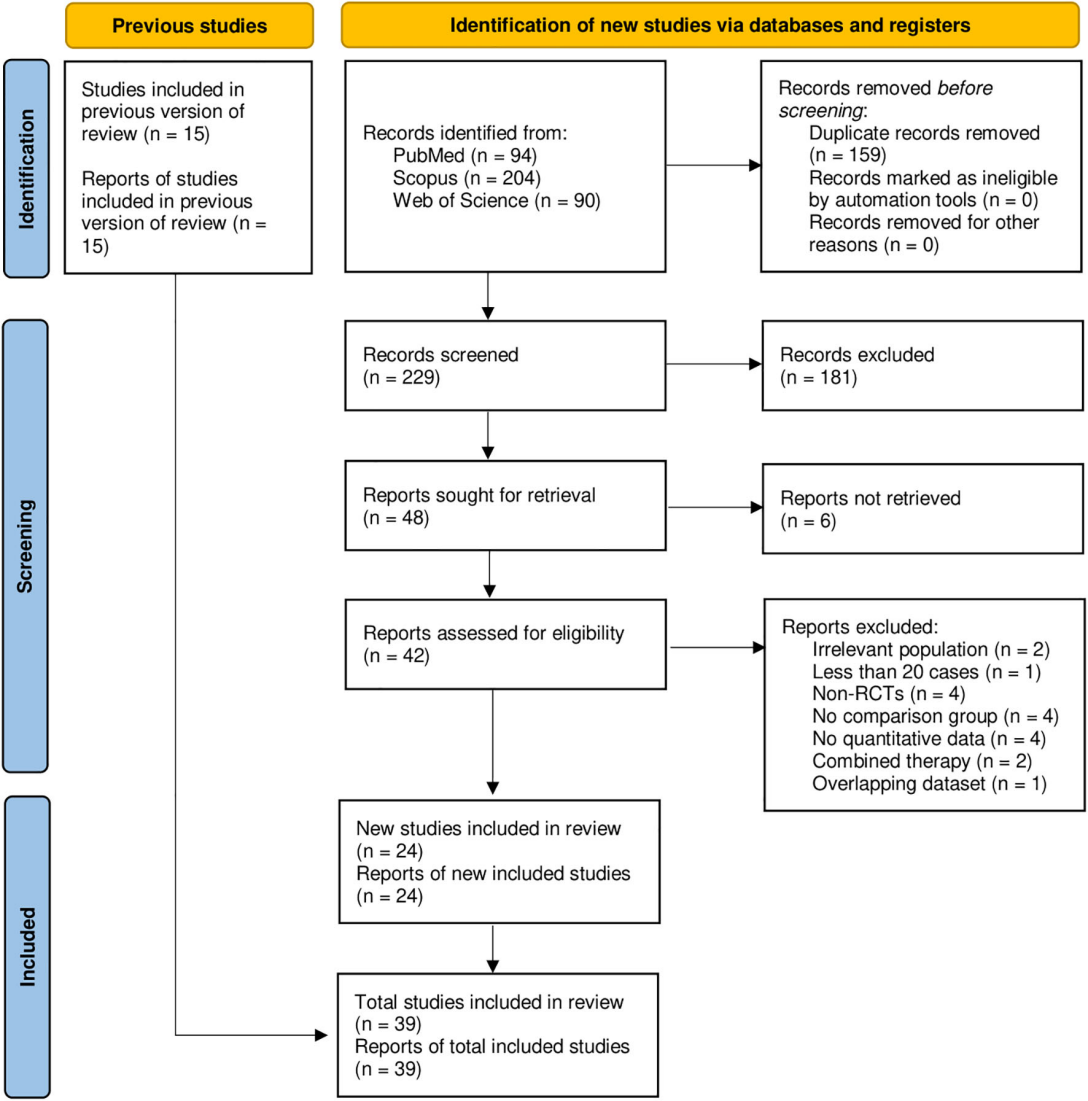
A PRISMA flow diagram showing the results of the database search.

The electronic search yielded 229 records after removal of 159 duplicates (**Figure 1**). Titles and abstracts were screened, resulting in the exclusion of 181 records. Forty-eight reports were sought for full-text retrieval, of which six could not be obtained. Forty-two full-text articles were assessed for eligibility. Twenty-two reports were excluded for the following reasons: irrelevant population (n=2), sample size fewer than 20 participants (n=1), non-randomized study design (n=4), absence of a comparator group (n=4), lack of extractable quantitative data (n=4), overlapping dataset (n = 1), and use of combined therapy that precluded isolation of the probiotic effect (n=2). Twenty-four newly identified randomized controlled trials met the inclusion criteria and were incorporated into the updated review. When combined with the 15 eligible trials from the previous version of the review (9), a total of 39 trials were included in the final analysis (4, 10–14, 21–53).

### 3.2 Baseline characteristics of included trials

A summary of included trials’ baseline characteristics is provided in **Table 1**. Most evidence came from Japan (10 RCTs) followed by China (seven trials), and Iran (three trials), respectively. Other trials spanned over 14 other countries. Overall, 4094 participants were examined, of whom 2053 were assigned to the probiotics group and 2041 to the placebo group. Data on included bacteria and probiotic dose (CFU/day) and follow-up period are presented in **Table 1**.

**Table 1:** Baseline characteristics of included randomized controlled trials.

Study ID	Country	Sample size		Age		Male (n)		Condition	Bacteria	CFU/day	FU
		Intervention	Placebo	Intervention	Placebo	Intervention	Placebo				
Ahmed 2025 (21)	India	50	49	–	–	–	–	healthy	*L. rhamnosus*	10B CFU	12-week
Askarpour 2025 (22)	Iran	40	41	33.46 (5.49)	33.42 (5.52)	all sample females		POS	*B. animalis and L. acidophilus*	≥10^6 CFU	4, 8-week
Baião 2022 (23)	UK	24	25	27.94(6.99)	29.64 (10.52)	13	13	healthy	*B. subtilis, B. bifidum, B. breve, B. infantis, B. longum, L. acidophilus, L. delbrueckii ssp. bulgaricus, L. casei, L. plantarum, L. rhamnosus, L. helveticus, L. salivarius, L. lactis ssp, S. thermophilus*	2x10 CFU	4-week
Du 2025 (12)	China	25	25	66.7 (9.1)	64.8 (8.7)	18	12	PD	*B. licheniformis*	2x10^9 CFU	12-week
Freijy 2023 (28)	New Zealand	29	27	23.4(10.2)	25.5 (4.92)	3	2	healthy	*B. bifidum; B. animalis; B. longum; L. acidophilus; L. helveticus; L. casei; L. plantarum; L. rhamnosus*	12 x10^9 CFU	8-week
Guan 2025 (10)	China	59	57	24.4 (2.2)	24.4 (2.3)	15	14	healthy	*L. paracasei*	6 × 10^10 CFU	2-week
Grant 2025 - 1mil CFU (11)	USA	27	31	–	–	–	–	self-reported mild to moderate anxiety	*Lactobacillus* strains	1x10^9 CFU	2, 4, 6-week
Grant 2025 - 27.5mil CFU (11)		25	31	–	–	–	–	*Lactobacillus* strains	27.5x 10^9 CFU	6-week
Jangpour 2024 (29)	Iran	28	24	47.19(12.63)	22.5 (1.75)	16	14	end-stage renal disease	*L. rhamnosus, L. Helveticus, L. casei, B. lactis, L. acidophilus, B. breve, L. bulgaricus, B. Langum, L. plantarum, B. bifidum, L. garii, and S. thermophilus*	–	4, 8-week
Kerksick 2024 (31)	USA	35	35	29.7 (9)	32.3 (10)	–	–	healthy	*L. fermentum, L. rhamnosus, L. plantarum, B. longum*	1x10^9 CFU	2, 4, 6, 9-week
Lan 2023 (4)	China	20	20	38.95 (10.59)	36.55 (11.31)	8	6	sleep disorder	*B. breve*	5x10^9 CFU	4-week
Li 2024 - 1mil CFU (34)	China	37	34	25.17 (2.78)	25.02 (4.32)	8	9	healthy	*L. paracasei*	1x10^9 CFU	4-week
Li 2024 - 5mil FU (34)		33	34	24.78 (3.02)	25.02 (4.32)	7	9	healthy	*L. paracasei*	5x10^9 CFU	4-week
Liu 2025 (35)	China	52	49	19.9 (1.3)	19.9 (1.4)	20	15	healthy	*B. animalis*	1x10^9 CFU	4, 8-week
Mäkelä 2023 (36)	UK	93	97	22.67 (2.56)	23.18 (2.69)	41	44	healthy	*L. paracasei*	1.56 × 10^10 CFU	10-week
Mörkl 2025 – Depressed (38)	Ireland	20	20	32.65 (8.83)	37.5 (14.73)	4	5	depression	*B. bifidum, B. lactis, L. acidophilus, L. casei, L. paracasei*	1.5 × 10^10 CFU	1, 4, 12-week
Mörkl 2025 – Healthy (38)		23	23	35.30 (10.10)	37.13 (14.68)	12	6	healthy
Murakami 2024 (14)	Japan	61	65	46.1 (7)	46.7(7.3)	32	30	healthy	*B. adolescentis*	1.0 × 10^11 CFU	4-week
Mutoh 2023 (39)	Japan	23	17	20.7(0.4)	20.9(0.5)	5	5	healthy	*L. helveticus*	5×10^9 CFU	2, 4-week
Mutoh 2024 (13)	Japan	15	15	46.3(11.6)	47.9(11.6)	8	8	healthy	*B. breve*	1 × 10^10 CFU	1-6-week
Önning 2023 (41)	Sweden	65	64	35.5(7.75)	34.9(7.25)	23	21	healthy	*L. plantarum*	10B CFU)	12-week
Othman 2023 (24)	Tunisia	15	15	–	–	–	–	obese	*L. helveticus*	1x10^9 CFU	4-week
Patterson 2024 (43)	Ireland	45	45	32.43(7.91)	30.13(8.41)	10	15	healthy	*B. longum*	1 × 10^9 CFU	4, 8-week
Rode 2025 – Encapsulated (45)	Sweden	28	29	69.1 (5)	69.5(5.2)	10	9	older adults	*L. rhamnosus*	6x10^9 CFU	3, 6-week
Rode 2025 - Non-encapsulated (45)		30	29	68.5 (4.9)	69.5(5.2)	10	9
Tian 2024 (49)	China	20	19	37.40 (14.31)	41.79 (17.81)	7	5	depressive disorder	*P. acidilactici*	1 × 10^9 CFU	4-week
Wu 2022 (51)	Taiwan	33	32	35.39(11.19)	36.24(9.2)	1	0	highly stressed employees	*L. paracasei*	10 billion HK-PS23 cells.	8-week
Zhu 2023 (53)	China	30	30	22.5(0.25)	22.3(0.25)	15	15	anxiety and depression	*L. plantarum JYLP-326*	1.5 × 10^10 CFU	3-week
Diop (2008) (27)	France	33	31	38 (11)	–	–	–	healthy	*L. acidophilus, B. longum*	3 x 10^9	3-week
Takada (2018) (48)	Japan	48	46	22.8(0.2)	22.6(0.2)	27	28	healthy	*L. casei*	1 X 10^11	6, 8, 11 weeks
Yoshikawa (2018) (52)	Japan	103	101	–	–	27	30	healthy	*L. brevis*	2.5 X 10^10	4-week
Marotta (2019) (37)	Australia	18	15	21.6(2.2)	21.7(2.2)	11	10	healthy	*L. fermentum, L. rhamnosus, L. plantarum, B. longum*	4x10^9	6-week
Nishida (2019) (40)	Japan	31	29	24.9(0.5)	25.3(0.6)	21	20	healthy	*L. gasseri*	1x10^10	12, 24 weeks
Kanesugi (2020) (30)	Japan	20	20	43.6(8.7)		16	16	healthy	*S. cerevisiae*	3x10^10	4-week
Patterson (2020) (42)	Japan	55	59	23.7(4.3)	23.3(4.2)	26	30	healthy	*L. paracasei*	1.75x10^10	5-week
Valle (2021) (50)	Brazil	32	33	19.7 ± 1.3	19.5 ± 1.2	20	19	healthy	*L. acidophilus*	1.7x10^11	4-week
Calgaro (2021) (26)	Italy	18	14	–	–	–	–	healthy	*L. fermentum, L. rhamnosus, L. plantarum, B. longum*	4x10^9	6-week
Kinoshita (2021) (32)	Japan	479	482	38(11)	–	0	0	healthy	*L. delbrueckii*	1.12x10^6	16-week
Lee (2021) (33)	Korea	63	59	38.9 ± 10.9	37.6 ± 11.0	18	21	healthy	*L. paracasei*	6x10^6	4-week
Calandre (2021) (25)	Spain	53	53	56(7.5)	55.5(8.6)	–	–	fibromyalgia	*S. thermophilus, B. breve*, *B. animalis*, *L. acidophilus*, *L. plantarum*, *L. paracasei, L. helveticus*	4x10^11	12-week
Segawa (2021) (46)	Japan	75	77	42.6(10.5)	42.6(10.1)	–	–	healthy	*L. paracasei*	6x10^10	12-week
Quero (2021) (44)	Spain	7	7	23(2.1)	24.3(3.9)	–	–	healthy	*B. lactis, L. rhamnosus*, *B. longum*	1 x10^9	4-week
Shafie (2022) (47)	Iran	33	33	51.8(2.3)	52.4(2.4)	0	0	healthy	*B. lactis, L. acidophilus*	2 x10^10	6-week

USA, United States of America; UK, United Kingdom; n, number of patients; FU, follow-up; CFU, colony-forming unit; POS, polycystic ovary syndrome; PD, Parkinson disease.

### 3.3 Risk of bias

**Figure 2:**
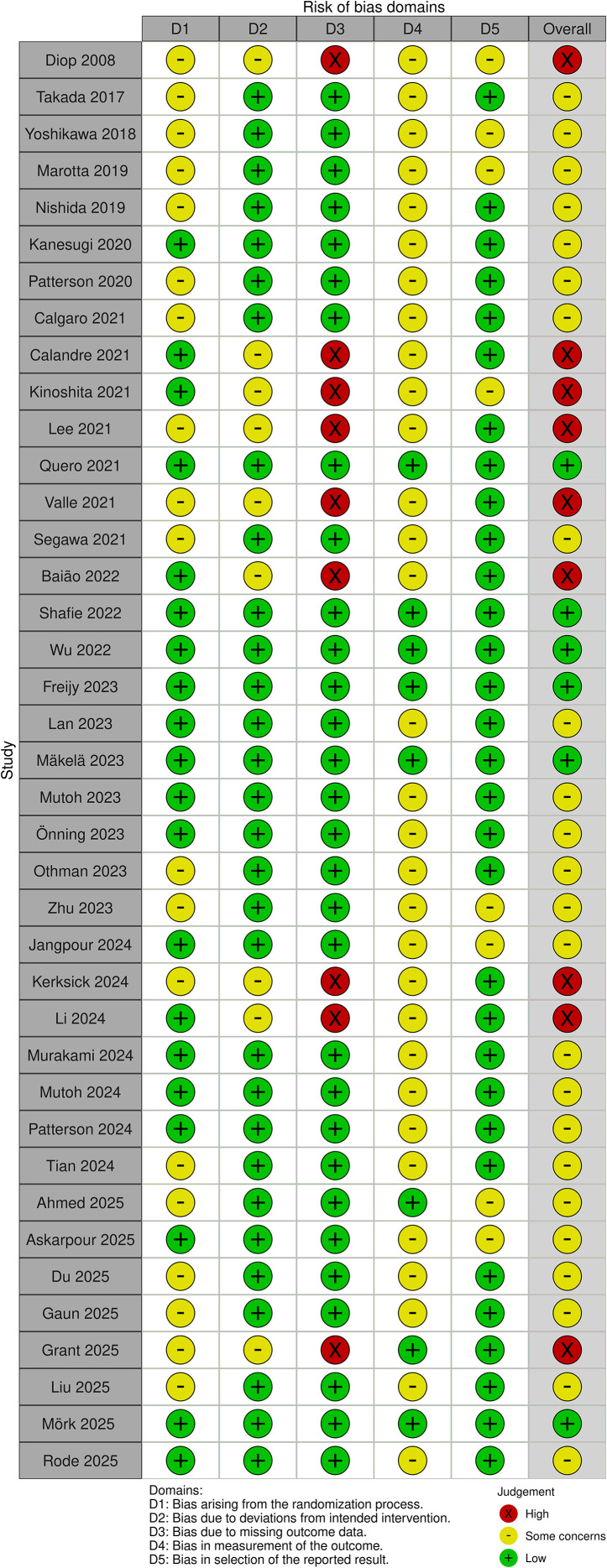
An illustration showing the risk of bias of included randomized controlled trials.

An illustration of the risk of bias of included trials is provided in **Figure 2**. Overall, six trials had low risk of bias, 24 trials had some concerns, and nine trials had high risk of bias. The concerns were majorly attributed to the lack of a detailed randomization protocol or inadequacy of selective reporting (lack of a pre-determined protocol).

### 3.4 Subjective sleep quality (PSQI)

**Figure 3:**
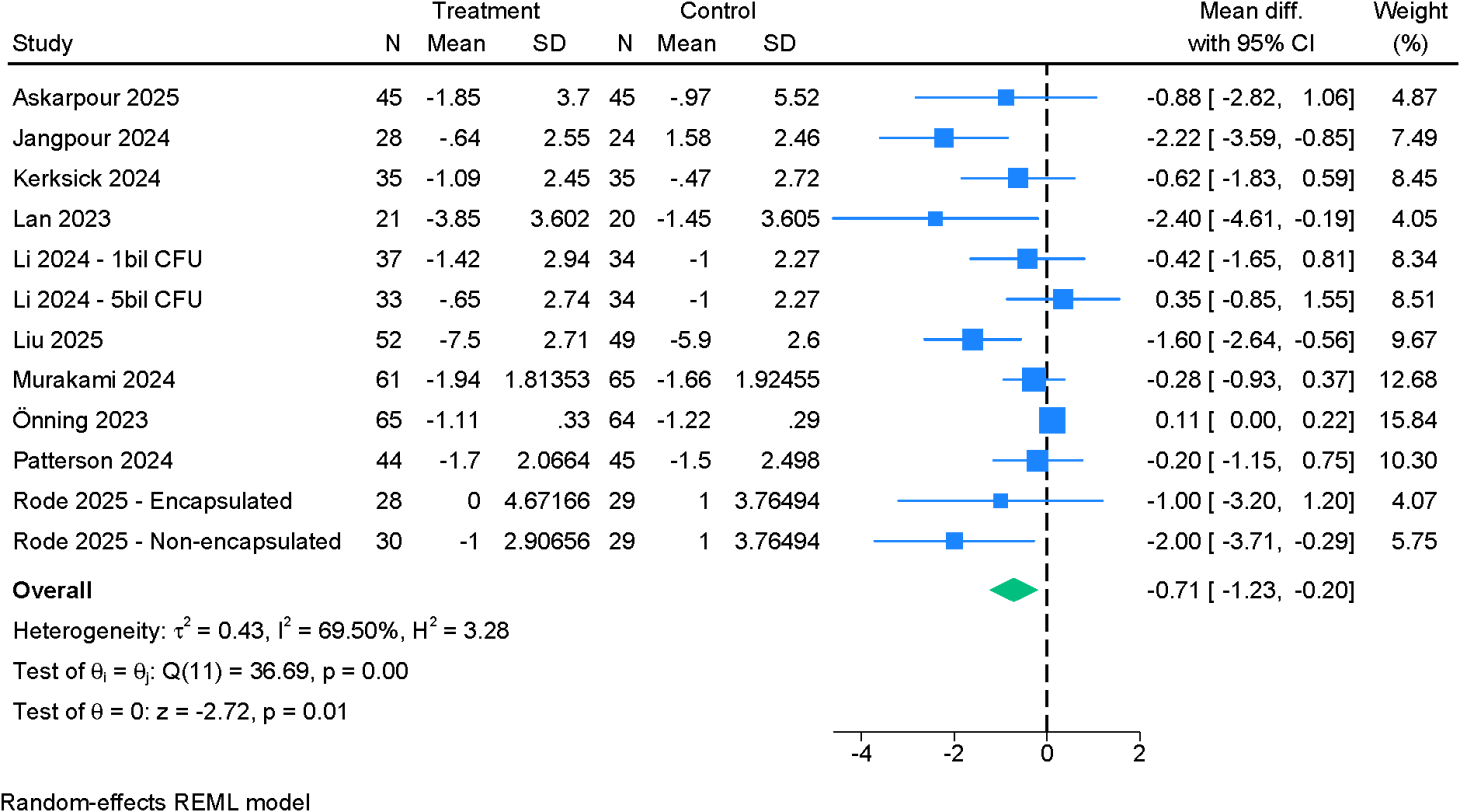
Forest plot showing the difference in mean change in PSQI (global score) between probiotics and placebo.

Ten randomized controlled trials contributed data to the primary outcome of change in PSQI global score (**Figure 3**). Probiotic supplementation resulted in a statistically significant improvement compared with placebo (MD –0.71; 95% CI –1.23 to –0.20; I²=69.5%). Sensitivity analyses did not materially alter the magnitude or direction of effect, although Egger’s test indicated evidence of small-study effects (p=0.0009). The certainty of evidence for this outcome was rated as very low (**Table 2**).

**Table 2:** A summary of the meta-analytic estimates of conducted analyses.

Outcome	Studies	MD (95% CI)	I^2^	Sensitivity	Egger’s (P-value)	GRADE
PSQI (global score)	10 RCTs	-0.71 (-1.23, -0.20)	69.50%	non-sign.	0.0009	Very low
PSQI (sleep quality)	6 RCTs	-0.14 (-0.35, 0.08)	79.48%	non-sign.	N/A	Low
PSQI (daytime dysfunction)	5 RCTs	-0.08 (-0.11, -0.05)	0%	N/A	N/A	Moderate
PSQI (sleep disturbance)	5 RCTs	-0.05 (-0.11, 0.02)	24.53%	N/A	N/A	Moderate
PSQI (sleep time)	5 RCTs	0.11 (0.01, 0.21)	21.13%	N/A	N/A	Moderate
PSQI (sleep efficiency)	4 RCTs	-0.21 (-0.44, 0.02)	79.38%	LOO: -0.31 (-0.55, -0.07)	N/A	Low
PSQI (sleep latency)	5 RCTs	-0.13 (-0.44, 0.19)	75.16%	non-sign.	N/A	Low
PSQI (sleep medications)	3 RCTs	0.07 (-0.02, 0.16)	0%	N/A	N/A	Moderate
REM (sleep efficiency)	3 RCTs	1.64 (-0.35, 3.64)	28.32%	N/A	N/A	Moderate
ISI/AIS score*	7 RCTs	0.17 (-1.15, 1.49)	98.14%	LOO: -0.44 (-0.74, -0.14)	N/A	Low
Total sleep time (mins)	3 RCTs	13.82 (1.15, 26.49)	0%	N/A	N/A	Moderate
Total wakefulness (mins)	2 RCTs	5.15 (0.43, 9.87)	0%	N/A	N/A	Moderate
OSA-MA Factor 1	4 RCTs	1.07 (0.40, 1.75)	0%	N/A	N/A	Moderate
OSA-MA Factor 2	4 RCTs	0.19 (-0.83, 1.21)	48.02%	N/A	N/A	Low
OSA-MA Factor 3	4 RCTs	-0.44 (-1.57, 0.70)	11.58%	N/A	N/A	Low
OSA-MA Factor 4	4 RCTs	1.18 (0.42, 1.94)	0%	N/A	N/A	Moderate
OSA-MA Factor 5	4 RCTs	0.11 (-0.77, 0.99)	0%	N/A	N/A	Moderate

*For this outcome, the Cohen’s D (standardized mean difference) was calculated to account for the variable measurement tools. Factor 1: Sleepiness on rising; Factor 2: Initiation and maintenance of sleep; Factor 3: Frequent dreaming; Factor 4: Refreshed on rising; Factor 5: Sleep length. MD, mean difference; RCT, randomized controlled trial; I2, a measure of statistical heterogeneity (significant heterogeneity is defined >50%); N/A, not applicable (no significant heterogeneity to warrant sensitivity analysis); LOO, leave-one-out sensitivity analysis; sign., significant; CI, confidence interval; PSQI, Pittsburgh Sleep Quality Index; ISI, Insomnia Severity Index; AIS, Athens Insomnia Scale; OSA-MA, OSA Sleep Inventory MA version; REM, rapid eye movement.

**Figure 4:**
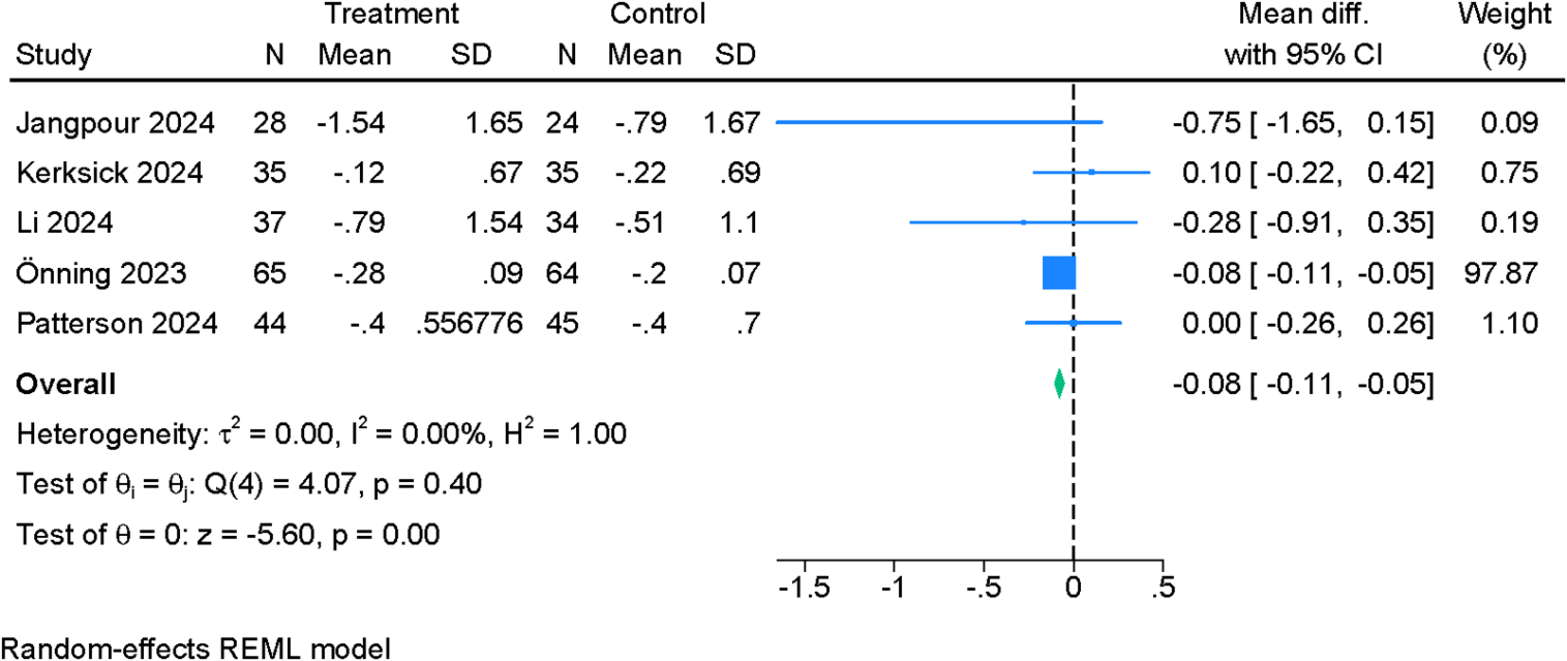
Forest plot showing the difference in mean change in PSQI (daytime dysfunction) between probiotics and placebo.

**Figure 5:**
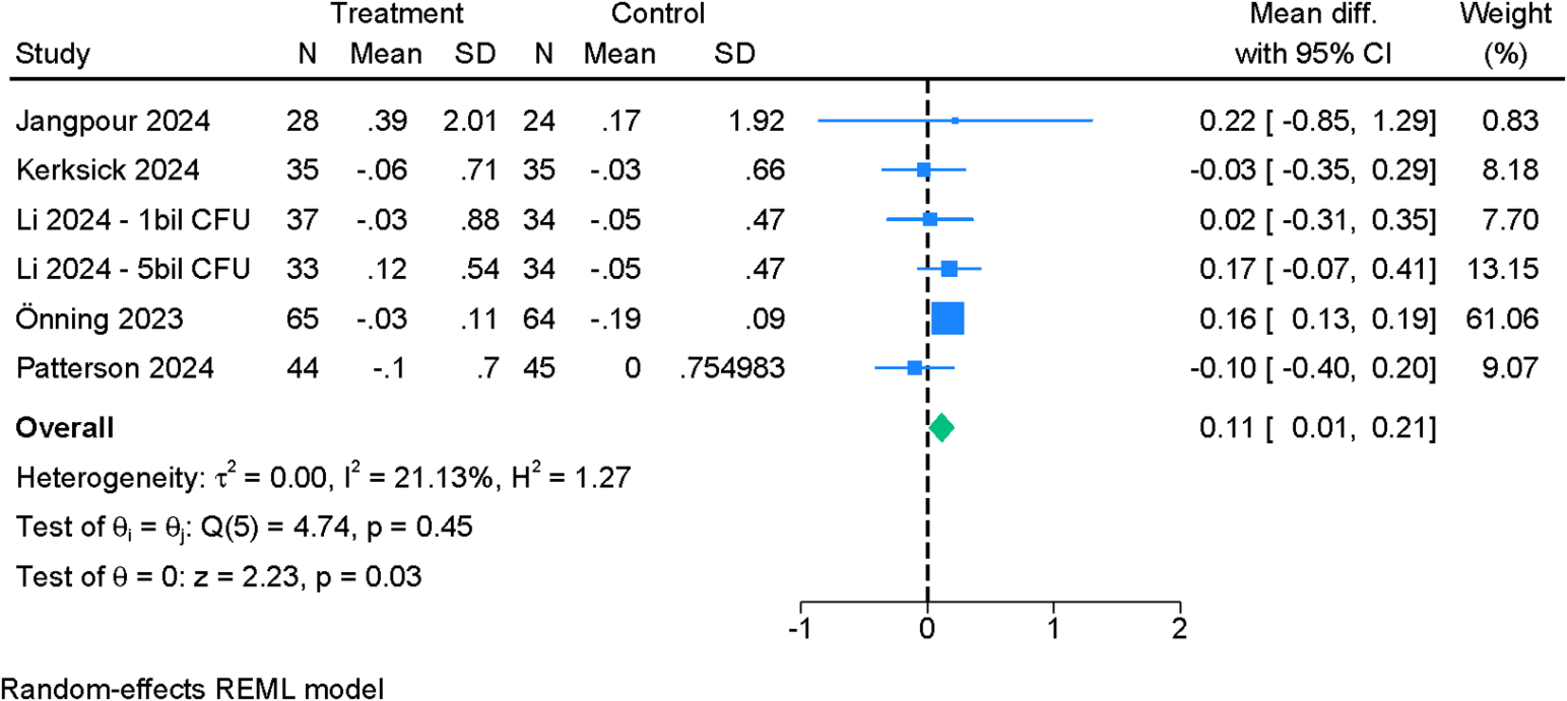
Forest plot showing the difference in mean change in PSQI (sleep time) between probiotics and placebo.

Across PSQI subdomains, the effects of probiotics were domain-specific. Daytime dysfunction (**Figure 4**) demonstrated a small but statistically robust improvement (MD –0.08; 95% CI –0.11 to –0.05; I²=0%). Sleep time (**Figure 5**) also increased modestly (MD 0.11; 95% CI 0.01 to 0.21; I²=21.13%). In contrast, sleep quality (**Supplementary Figure 1**) (MD –0.14; 95% CI –0.35 to 0.08; I²=79.48%), sleep disturbance (**Supplementary Figure 2**) (MD –0.05; 95% CI –0.11 to 0.02; I²=24.53%), sleep latency (**Supplementary Figure 5**) (MD –0.13; 95% CI –0.44 to 0.19; I²=75.16%), and sleep efficiency (**Supplementary Figure 3**) (MD –0.21; 95% CI –0.44 to 0.02; I²=79.38%) did not show significant between-group differences. However, leave-one-out analysis for sleep efficiency identified a significant improvement when one influential study was excluded (MD –0.31; 95% CI –0.55 to –0.07) (**Supplementary Figure 4**). Use of sleep medications was unchanged between groups (**Supplementary Figure 6**). Certainty of evidence ranged from moderate (daytime dysfunction, sleep disturbance, sleep time, sleep medication use) to low (sleep quality, sleep efficiency, sleep latency) (**Table 2**).

### 3.5 Insomnia severity

Seven RCTs assessing insomnia severity through various instruments (ISI or AIS) were synthesized using standardized mean differences (**Supplementary Figure 8**). The pooled effect was not statistically significant (SMD 0.17; 95% CI –1.15 to 1.49; I²=98.14%). Considerable heterogeneity was observed across studies, reflecting both methodological and clinical variability. In leave-one-out analysis, exclusion of a single influential trial yielded a significant pooled reduction in insomnia severity (SMD –0.44; 95% CI –0.74 to –0.14) (**Supplementary Figure 9**). Overall certainty of evidence for this outcome was rated as low.

### 3.6 Objective sleep parameters

**Figure 6:**
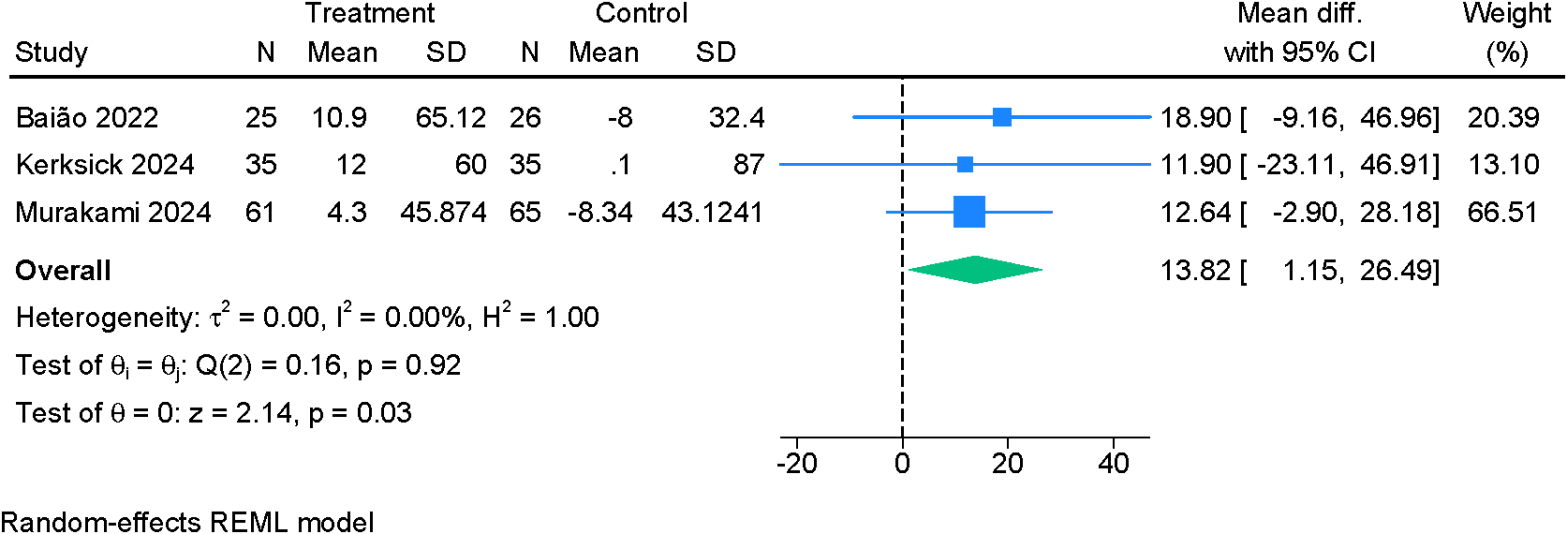
Forest plot showing the difference in mean change in total sleep time (in minutes) between probiotics and placebo.

**Figure 7:**
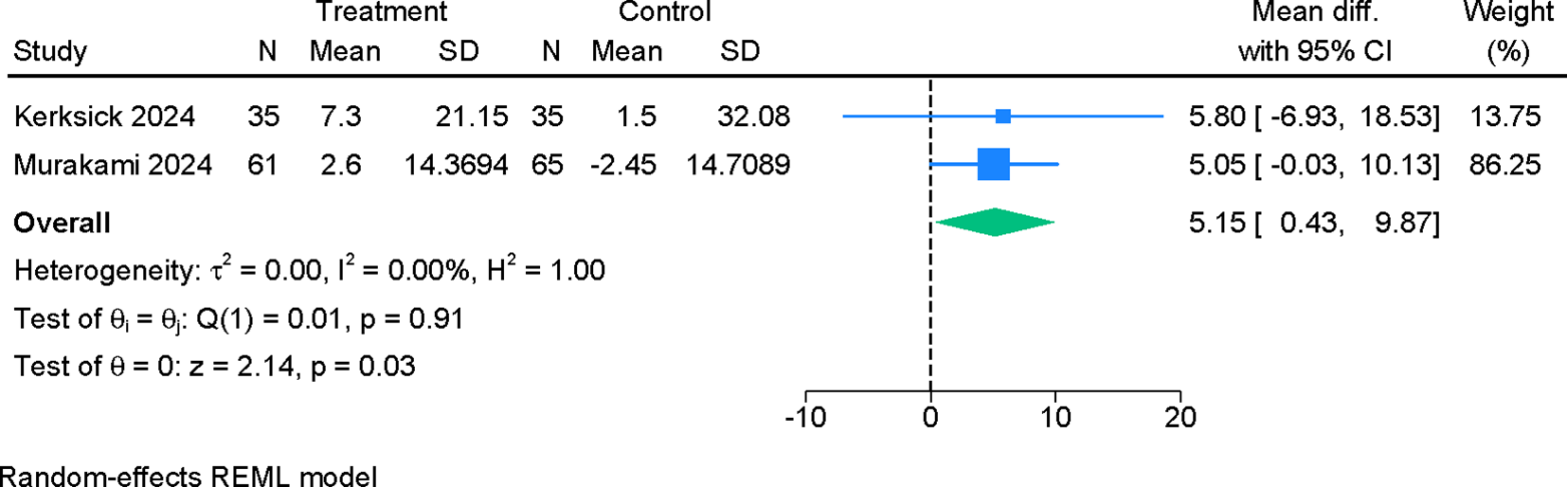
Forest plot showing the difference in mean change in total wakefulness time (in minutes) between probiotics and placebo.

Three studies evaluating REM-associated sleep efficiency found no significant effect of probiotic supplementation (MD 1.64; 95% CI –0.35 to 3.64; I²=28.32%) (**Supplementary Figure 7**). In contrast, probiotics led to a significant prolongation of total sleep time (MD 13.82 minutes; 95% CI 1.15 to 26.49; I²=0%) (**Figure 6**). Minutes awake during the sleep period also differed significantly between groups, with probiotics associated with a modest reduction (MD 5.15 minutes; 95% CI 0.43 to 9.87; I²=0%) (**Figure 7**). Certainty of evidence for objective outcomes was assessed as moderate (**Table 2**).

### 3.7 OSA-MA sleep inventory factors

**Figure 8:**
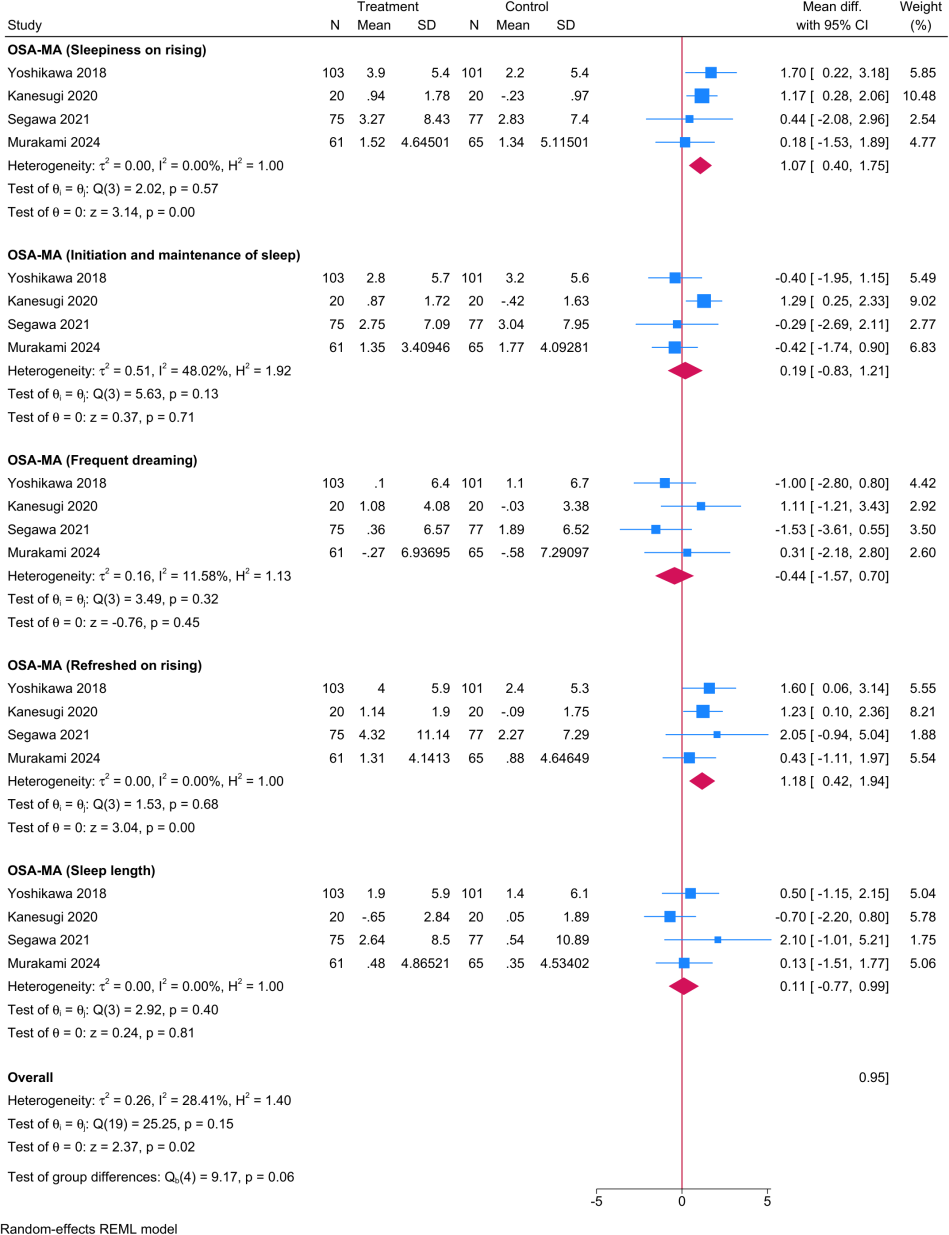
Forest plot showing the difference in mean change in OSA – Factor 1 between probiotics and placebo.

Four trials reported outcomes using the OSA Sleep Inventory MA version (**Figure 8**). Probiotics produced significant improvements in sleepiness on rising (Factor 1; MD 1.07; 95% CI 0.40 to 1.75; I²=0%) and in the sense of being refreshed upon awakening (Factor 4; MD 1.18; 95% CI 0.42 to 1.94; I²=0%). No significant differences were observed for initiation and maintenance of sleep (Factor 2; MD 0.19; 95% CI –0.83 to 1.21; I²=48.02%), frequent dreaming (Factor 3; MD –0.44; 95% CI –1.57 to 0.70; I²=11.58%), or perceived sleep length (Factor 5; MD 0.11; 95% CI –0.77 to 0.99; I²=0%). Certainty of evidence ranged from moderate for Factors 1, 4, and 5 to low for Factors 2 and 3 (**Table 2**).

## 4 Discussion

In this updated meta-analysis, probiotic supplementation was associated with a modest but statistically significant improvement in global subjective sleep quality, as reflected by the PSQI global score. However, this finding was accompanied by moderate statistical heterogeneity (I² ≈ 70%), indicating variability in effect size across included trials. While the overall direction of effect consistently favored probiotics, the magnitude of benefit varied between studies, underscoring the need to interpret pooled estimates as average effects across heterogeneous settings rather than uniform effects applicable to all populations. Similar domain-specific benefits were observed for daytime dysfunction and sleep duration, as well as improvements in morning refreshment and reduced sleepiness on rising captured by selected OSA-MA factors. A limited subset of trials reporting objective sleep measures also showed modest gains: increased total sleep time and reduced minutes awake during sleep period under probiotic treatment compared with placebo.

Hence, the aggregate evidence suggests that probiotics may exert a small beneficial effect on subjective sleep quality, some aspects of sleep continuity and daytime functioning, and, potentially, on objective sleep duration. These effects, though modest, warrant consideration because probiotics offer a generally low-risk, widely accessible intervention in immunocompetent individuals. Notably, the magnitude of improvement observed for global subjective sleep quality was small. The pooled reduction in PSQI global score (MD –0.71) is substantially below commonly cited thresholds proposed for minimal clinically important difference, which have generally ranged from approximately 2 to 3 points in clinical populations. Similarly, while certain domain-specific PSQI subcomponents and OSA-MA factors showed statistically significant improvements, the absolute effect sizes were modest. These findings indicate that statistical significance does not necessarily equate to clinically meaningful benefit, and that the observed improvements may be most relevant at the population level or in individuals with mild sleep complaints rather than as a stand-alone therapeutic effect for clinically significant insomnia. It should be noted that the OSA Sleep Inventory MA assesses subjective dimensions of sleep and awakening (e.g., sleepiness on rising and refreshed feeling upon awakening) and does not directly quantify nocturnal wakefulness or wake after sleep onset; therefore, OSA-MA findings should not be interpreted as measures of night-time wakefulness.

Given the multidimensional structure of the OSA Sleep Inventory MA, differences in the direction and magnitude of effects across individual factors likely reflect domain-specific responsiveness rather than inconsistent or opposing treatment effects, with improvements observed primarily in factors related to awakening quality.

Our findings are broadly consistent with the conclusions of the most recent comprehensive systematic review by Ito et al. (9), conducted by an independent research group, which reported that probiotic supplementation may improve global sleep quality as assessed by PSQI and selected OSA-MA subscales. The present update retains the same underlying clinical question and core eligibility framework, while incorporating randomized trials published after the search end date of the prior review. As a result of the expanded evidence base, additional analyses using change-score data and a broader set of reported outcomes were feasible. These differences reflect the availability of new data rather than changes in the predefined research question or study eligibility criteria.

Second, we expanded the scope to include not only global sleep quality but also PSQI subdomains, objective sleep metrics, and morning alertness/refreshment (OSA-MA). This broader outcome repertoire allowed us to detect modest improvements in specific dimensions of sleep and daytime functioning that would likely be missed by analyses limited to global PSQI or insomnia severity only. Thus, while both reviews converge on a modest favorable association between probiotic supplementation and subjective sleep quality, the present update incorporates newly published trials and additional reported outcomes, allowing a more comprehensive synthesis of the current randomized evidence without altering the underlying clinical focus.

At the same time, other recent meta-analyses — including those combining probiotics with prebiotic or synbiotic interventions — have reported more mixed results. For example, a recent meta-synthesis concluded that while self-reported sleep quality sometimes improved, objective sleep parameters often did not, and the overall clinical significance remained unclear (54, 55). Thus, our results align with, but modestly advance beyond, the existing literature by affirming small but consistent benefits across multiple sleep dimensions.

The modest beneficial effects observed are biologically plausible in light of emerging evidence on the gut–brain axis. Gut microbiota regulates sleep-wake behavior via neuroimmune, neuroendocrine, and metabolic pathways (56–58). Animal and human data indicate that alterations in microbial composition influence circulating metabolites, immune signaling, and HPA-axis activity, which in turn affect central nervous system processes governing sleep architecture, stress response, and circadian regulation (35, 57, 59, 60). In humans, specific probiotic strains have been shown to modulate neurotransmitter precursors and reduce markers of systemic inflammation, factors that may favor enhanced sleep quality (7, 35). Nonetheless, given the current lack of standardization in probiotic interventions, direct extrapolation from mechanistic models to clinical sleep outcomes remains tentative.

### 4.1 Strengths and limitations

A major strength of this analysis is the use of change-score data (post–pre difference) for both probiotic and placebo groups. This design reduces bias associated with baseline differences and improves internal validity over analyses relying on end-of-treatment comparisons. Additionally, our broader inclusion criteria — encompassing a wide range of validated subjective outcomes (PSQI, OSA-MA), insomnia scales, and objective measures — allowed a more comprehensive assessment of probiotic effects on sleep. By grading the certainty of evidence for each outcome (using standard criteria), we provide transparent appraisal of confidence in the findings. Finally, by incorporating the latest trials, our review reflects the current state of the evidence and improves precision of pooled estimates.

Despite these strengths, several limitations constrain the interpretation of our findings. First, substantial statistical heterogeneity was observed for several key outcomes, including PSQI global score and insomnia severity, reflecting marked variability in effect magnitude across trials. Although random-effects models were used to account for between-study heterogeneity, high I² values indicate that pooled estimates should be interpreted as average effects rather than evidence of consistent benefit across all study settings. Sensitivity analyses suggested that, for some outcomes, statistical significance was influenced by a limited number of studies, highlighting the distinction between statistical significance and robustness across heterogeneous populations. Second, the overall number of trials contributing to certain outcomes—particularly objective sleep measures and OSA-MA subscales—remains limited, restricting precision and generalizability. In addition, the meta-analysis of wakefulness duration was based on only two trials, limiting the reliability and generalizability of this estimate; accordingly, this finding should be interpreted with particular caution.

Probiotic regimens differed markedly in strain composition, dosage, formulation (e.g., single strain vs multi-strain, CFU dose, delivery vehicle), duration of supplementation, and participant characteristics (healthy volunteers vs individuals with insomnia or other conditions). This heterogeneity reduces the ability to identify which specific probiotic regimens are effective, and precluded reliable subgroup analysis. From a methodological perspective, the degree of clinical and intervention heterogeneity raises the possibility that pooling highly diverse probiotic formulations and delivery modes may attenuate or obscure strain- or context-specific effects, suggesting that greater standardization and precision in future trials will be essential to reduce noise and improve signal detection.

Third, not all trials reported standard deviations of change-scores, necessitating variance imputations using assumed correlation coefficients; while such methods follow accepted meta-analytic practice, they introduce additional uncertainty into effect estimates. Fourth, the possibility of publication bias cannot be excluded — small studies with null or negative outcomes may remain unpublished, particularly given the relative novelty of probiotics–sleep research. Finally, the clinical significance of the observed effect sizes remains uncertain: while statistically significant, the magnitude of change (e.g., <1 point on PSQI) may not translate into perceptible improvement for patients.

### 4.2 Clinical implications

Given the favorable safety profile, widespread availability, and low cost of many probiotic products, the modest improvements identified in this study may have practical relevance as adjunctive measures, particularly for individuals with mild to moderate sleep disturbances, subclinical insomnia, or those seeking non-pharmacologic options. Clinicians may consider probiotics as part of a broader, multi-modal sleep hygiene and wellness strategy, recognizing that they are unlikely to replace first-line behavioral or pharmacologic therapies when clinically significant sleep disorders are present. Importantly, the potential for additive or synergistic effects when probiotics are combined with established treatments—such as cognitive behavioral therapy for insomnia or continuous positive airway pressure for obstructive sleep apnea—has not been evaluated in the available randomized trials and therefore cannot be inferred from the current evidence. Accordingly, it is premature to recommend probiotics as a first-line or disease-modifying intervention, and larger, well-powered trials with standardized probiotic regimens and longer follow-up are required before integration into routine sleep-management guidelines can be considered.

## 5 Conclusion

This updated systematic review and meta-analysis indicates that probiotic supplementation is associated with small but statistically significant improvements in subjective sleep quality, selected PSQI subdomains, and aspects of morning alertness and refreshment as assessed by the OSA Sleep Inventory MA. However, effect sizes are modest, clinical relevance remains uncertain, and the evidence base is constrained by substantial heterogeneity, limited numbers of high-quality trials for several outcomes, and possible publication bias. Findings related to objective sleep parameters should be interpreted with caution due to the small number of contributing studies. Overall, while probiotics may have a role as an adjunctive approach to improving sleep in selected populations, further rigorously designed, adequately powered randomized controlled trials using standardized probiotic formulations and outcome measures are required before firm clinical recommendations can be made.

## Data Availability

The original contributions presented in the study are included in the article/**Supplementary Material**. Further inquiries can be directed to the corresponding author.

## Supplementary material

Supplementary Figure 1 Forest plot showing the difference in mean change in PSQI (sleep quality) between probiotics and placebo.

Supplementary Figure 2 Forest plot showing the difference in mean change in PSQI (sleep disturbance) between probiotics and placebo.

Supplementary Figure 3 Forest plot showing the difference in mean change in PSQI (sleep efficiency) between probiotics and placebo.

Supplementary Figure 4 Leave-one-out sensitivity analysis of the difference in mean change in PSQI (sleep efficiency) between probiotics and placebo.

Supplementary Figure 5 Forest plot showing the difference in mean change in PSQI (sleep latency) between probiotics and placebo.

Supplementary Figure 6 Forest plot showing the difference in mean change in PSQI (sleep medications) between probiotics and placebo.

Supplementary Figure 7 Forest plot showing the difference in mean change in REM (sleep efficiency) between probiotics and placebo.

Supplementary Figure 8 Forest plot showing the difference in mean change in AIS/ISI score between probiotics and placebo.

Supplementary Figure 9 Leave-one-out sensitivity analysis of the difference in mean change in AIS/ISI score between probiotics and placebo.
