# Supplementary figures and images for: Effects of probiotic supplementation on subjective and objective sleep outcomes: an updated systematic review and meta-analysis of 39 randomized controlled trials
Source: Front Psychiatry. 2026 May 19;17:1769331. doi: 10.3389/fpsyt.2026.1769331 (PMC13226208; doi:10.3389/fpsyt.2026.1769331)

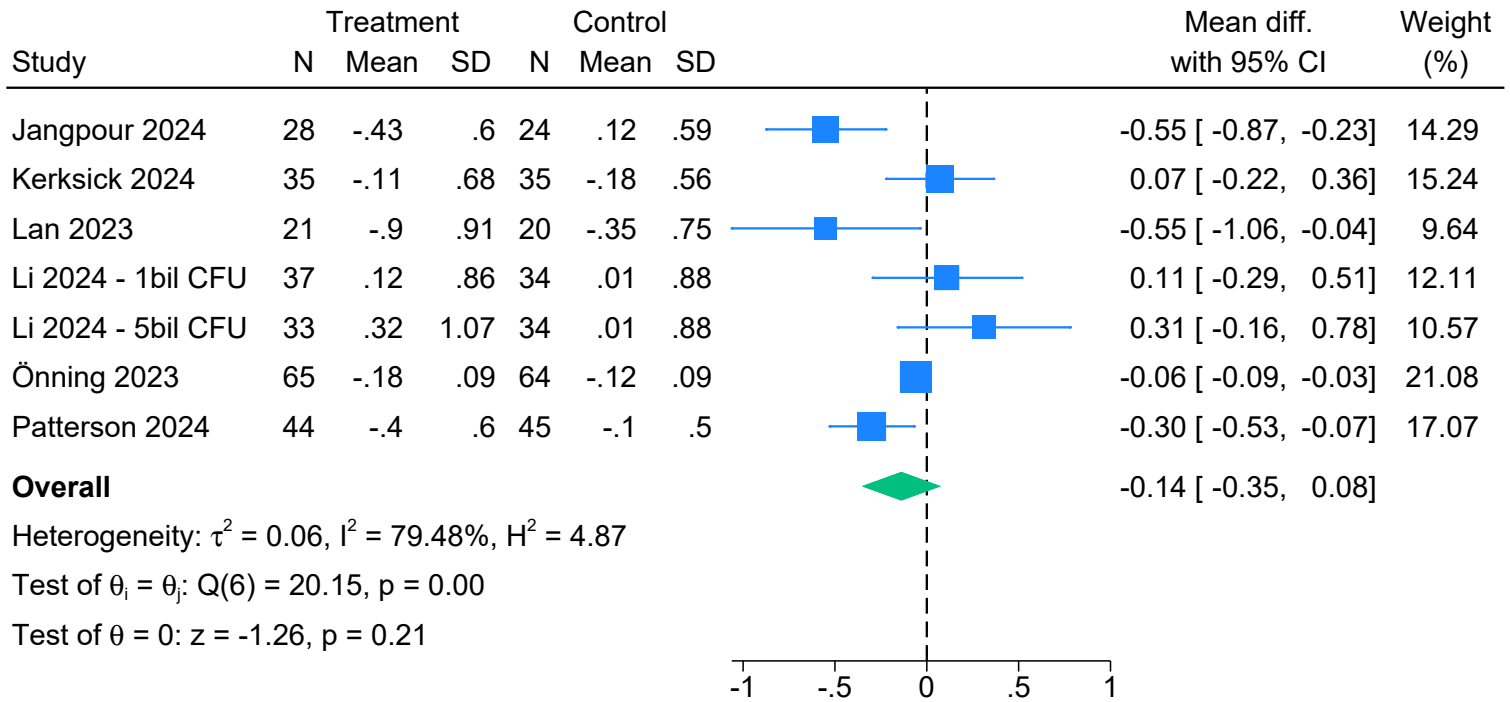

Random-effects REML model

Supplement: Supplementary Figure 1 — Forest plot showing the difference in mean change in PSQI (sleep quality) between probiotics and placebo. [file Image1.pdf]

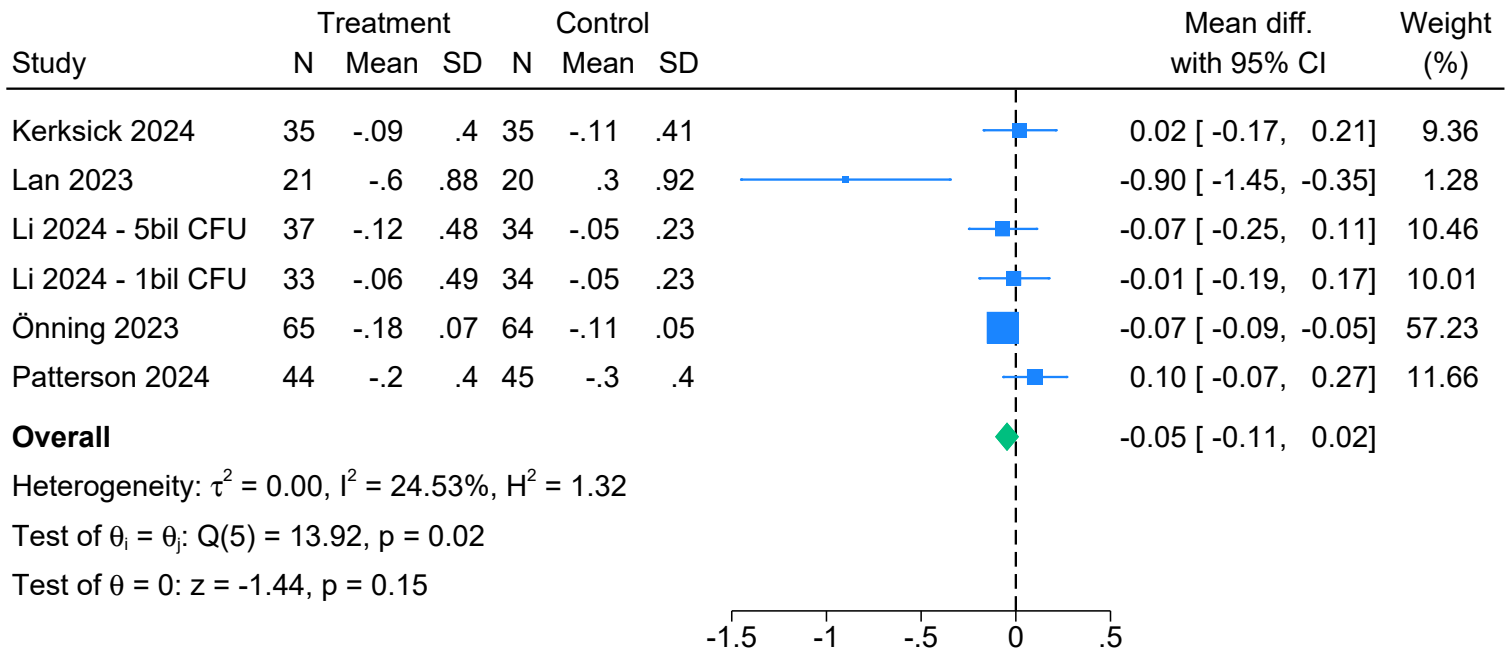

Random-effects REML model

Supplement: Supplementary Figure 2 — Forest plot showing the difference in mean change in PSQI (sleep disturbance) between probiotics and placebo. [file Image2.pdf]

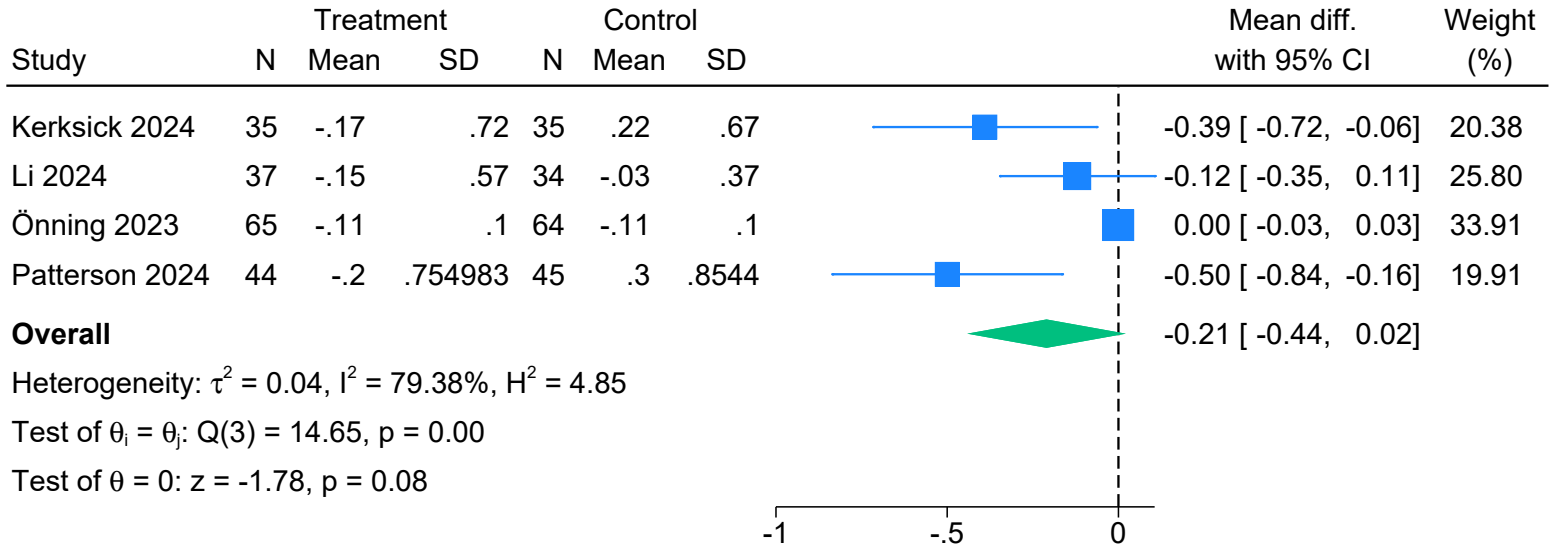

Random-effects REML model

Supplement: Supplementary Figure 3 — Forest plot showing the difference in mean change in PSQI (sleep efficiency) between probiotics and placebo. [file Image3.pdf]

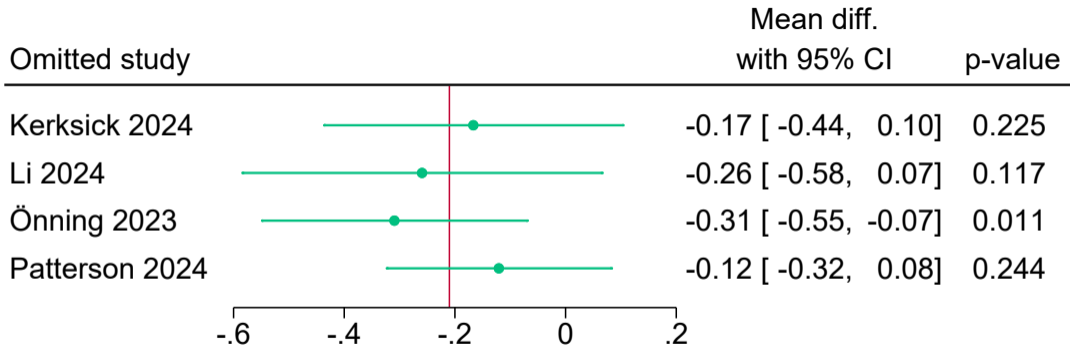

Random-effects REML model

Supplement: Supplementary Figure 4 — Leave-one-out sensitivity analysis of the difference in mean change in PSQI (sleep efficiency) between probiotics and placebo. [file Image4.pdf]

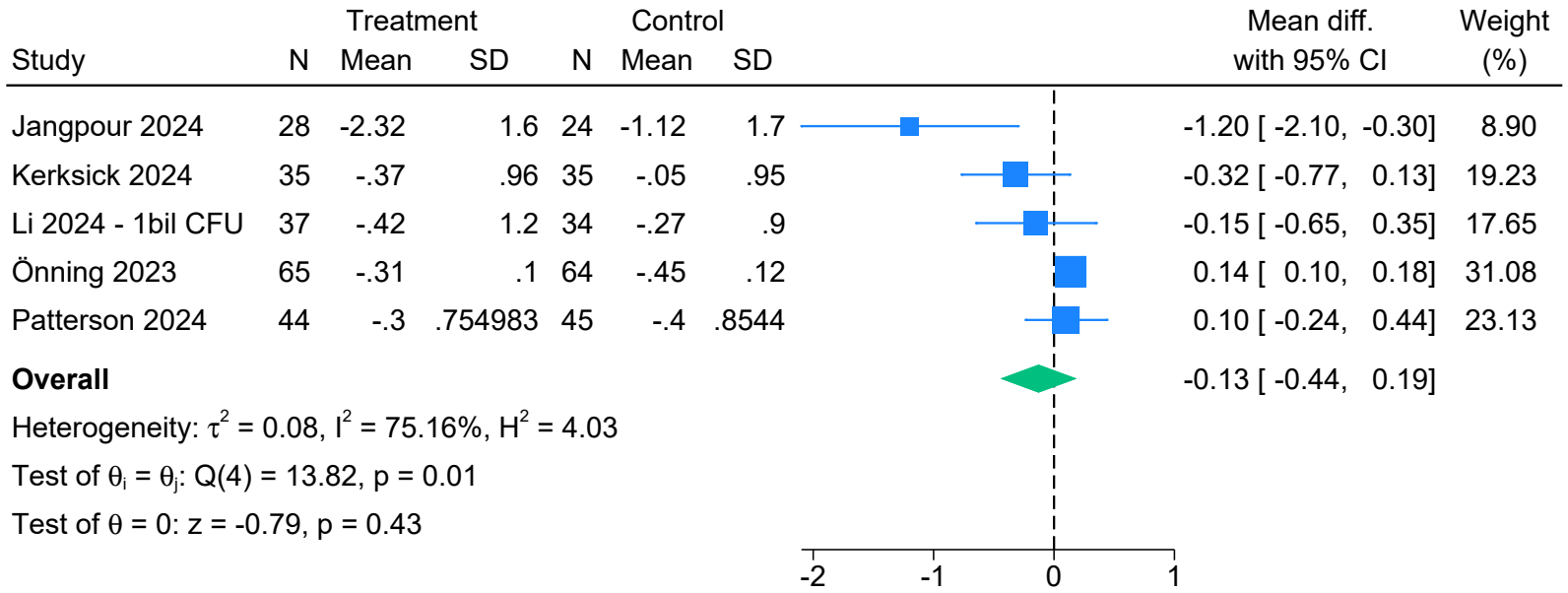

Random-effects REML model

Supplement: Supplementary Figure 5 — Forest plot showing the difference in mean change in PSQI (sleep latency) between probiotics and placebo. [file Image5.pdf]

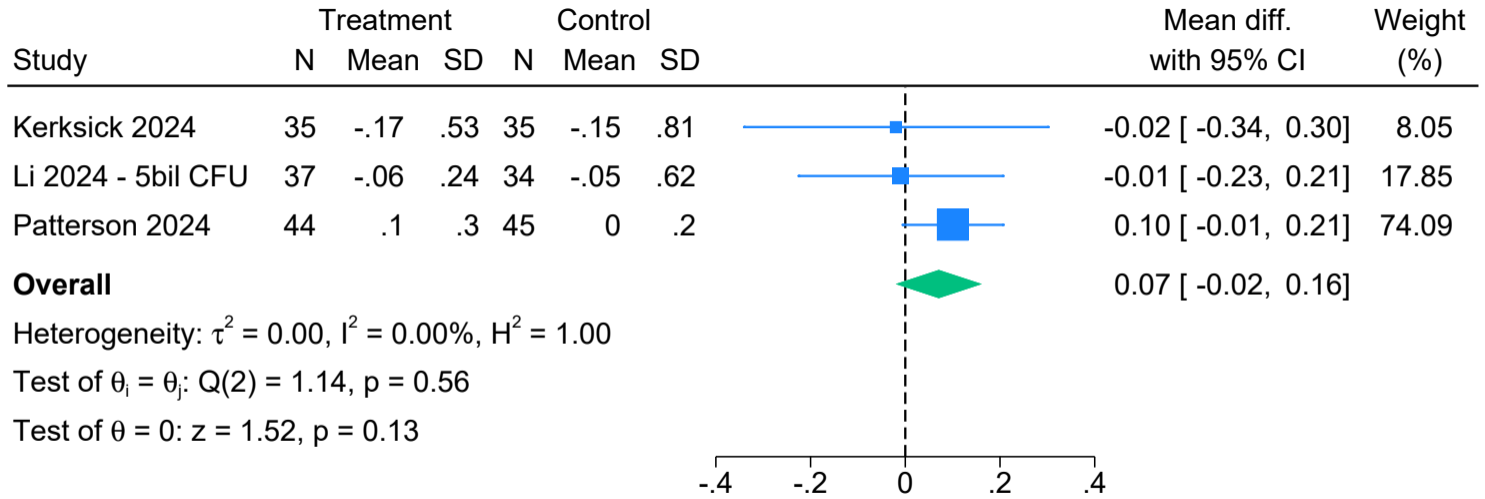

Random-effects REML model

Supplement: Supplementary Figure 6 — Forest plot showing the difference in mean change in PSQI (sleep medications) between probiotics and placebo. [file Image6.pdf]

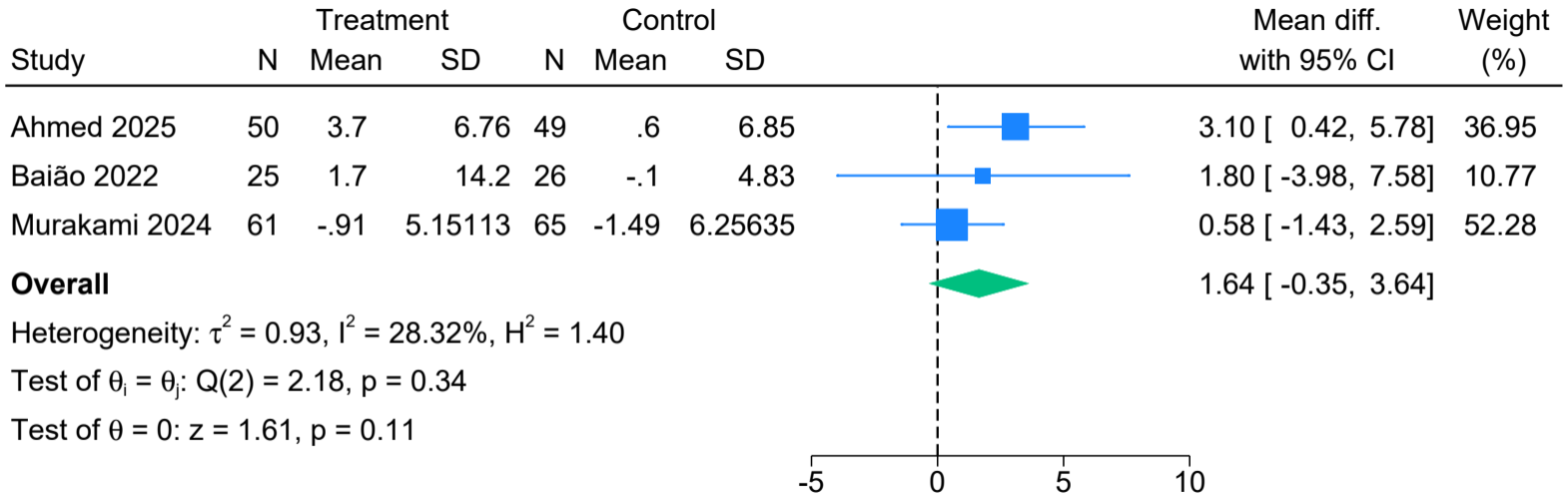

Random-effects REML model

Supplement: Supplementary Figure 7 — Forest plot showing the difference in mean change in REM (sleep efficiency) between probiotics and placebo. [file Image7.pdf]

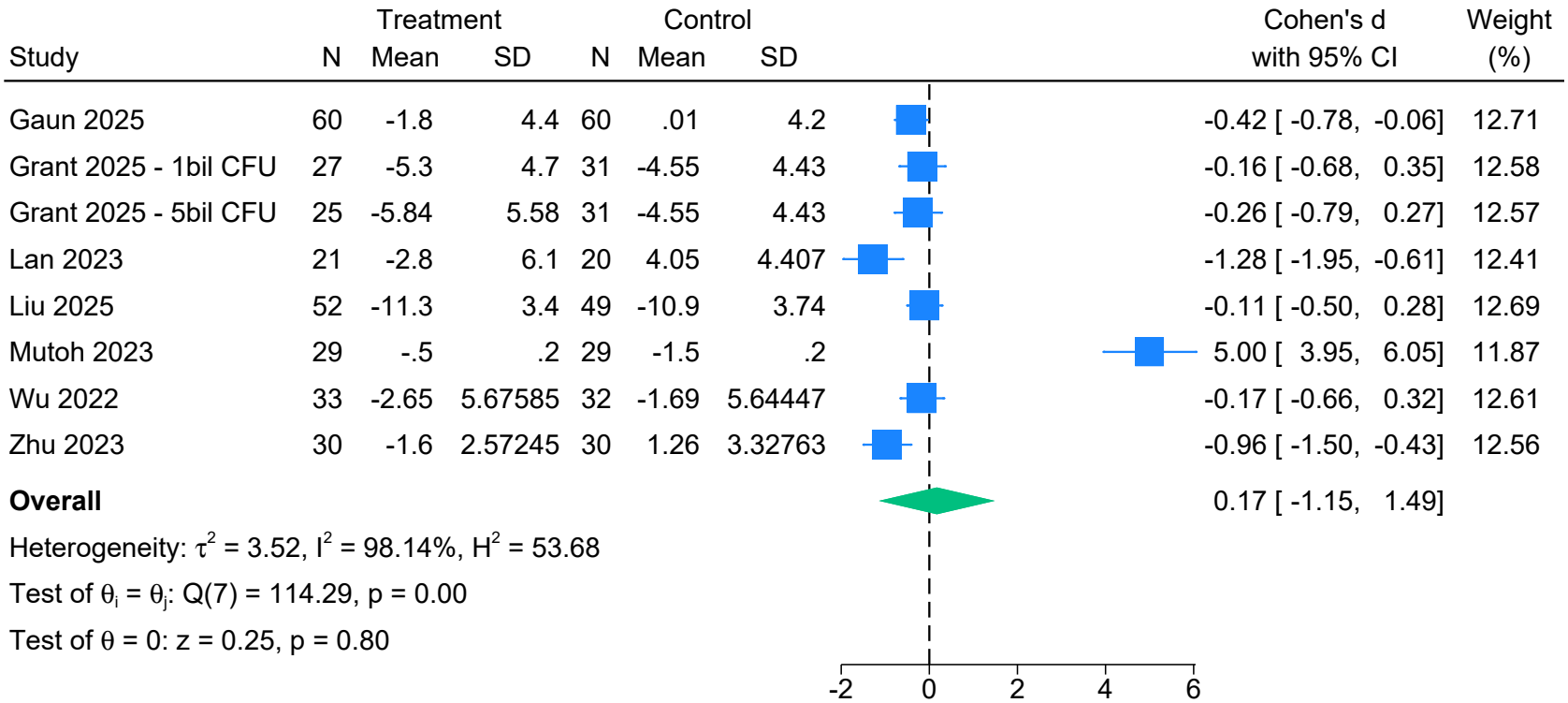

Random-effects REML model

Supplement: Supplementary Figure 8 — Forest plot showing the difference in mean change in AIS/ISI score between probiotics and placebo. [file Image8.pdf]

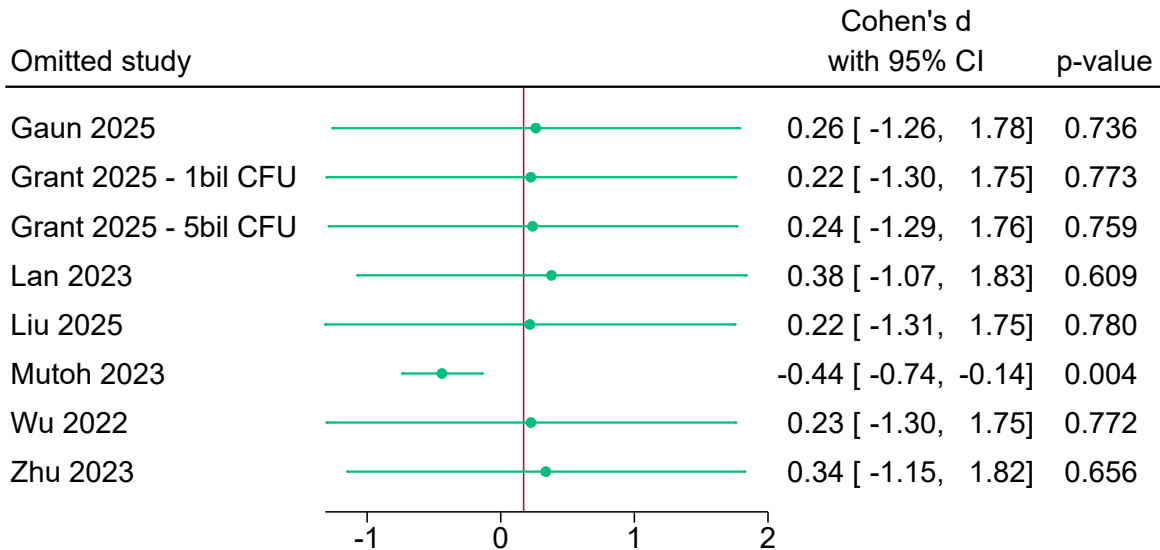

Random-effects REML model

Supplement: Supplementary Figure 9 — Leave-one-out sensitivity analysis of the difference in mean change in AIS/ISI score between probiotics and placebo. [file Image9.pdf]
