# Supplementary material for: Effects of probiotic supplementation on subjective and objective sleep outcomes: an updated systematic review and meta-analysis of 39 randomized controlled trials
Source: Front Psychiatry. 2026 May 19;17:1769331. doi: 10.3389/fpsyt.2026.1769331 (PMC13226208; doi:10.3389/fpsyt.2026.1769331)
Supplement: Supplementary file 10 [file Table1.docx]

**#0806: Effects of probiotics on sleep parameters: An updated systematic review AND meta-analysis of Randomized Controlled Trials**

Last review search date: March 31, 2022. **Date of updated search**: October 1^st^, 2025

**PubMed**

| No. | Search query | Results |
| --- | --- | --- |
| #1 | "probiotics"[MeSH Major Topic] OR "probiotic*"[tiab] OR "synbiotics"[MeSH Major Topic] OR "synbiotic*"[tiab] OR "paraprobiotic*"[tiab] OR "para psychobiotic*"[tiab] OR "psychobiotic*"[tiab] OR "lactobacillaceae"[MeSH Major Topic] OR "lactobacillaceae"[tiab] OR "Lacticaseibacillus"[tiab] OR "Lactobacillus"[tiab] OR "latilactobacillus sakei"[tiab] OR "levilactobacillus brevis"[tiab] OR "ligilactobacillus salivarius"[tiab] OR "Limosilactobacillus"[tiab] OR "Pediococcus"[tiab] OR "Bifidobacterium"[MeSH Major Topic] OR "Bifidobacterium"[tiab] OR "saccharomyces boulardii"[MeSH Major Topic] OR "saccharomyces boulardii"[tiab] | 102,294 |
| #2 | "sleep"[MeSH Terms] OR "sleep*"[tiab] | 294,689 |
| #3 | "placebos"[MeSH Terms] OR "placebo*"[tiab] OR "clinical trial"[Publication Type] OR "clinical trial"[tiab] OR "randomized controlled trial"[tiab] OR randomized[tiab] OR randomised[tiab] OR randomly[tiab] | 1,945,091 |
| #4 | #1 AND #2 AND #3 | 158 |
|  | Limit: March 31, 2022 >> October 1^st^, 2025 | 94 |

**Scopus**

| No. | Search query | Results |
| --- | --- | --- |
| #1 | TITLE-ABS-KEY ("probiotic*") OR TITLE-ABS-KEY ("synbiotic*") OR TITLE-ABS-KEY ("paraprobiotic*") OR TITLE-ABS-KEY ("para psychobiotic*") OR TITLE-ABS-KEY ("psychobiotic*") OR TITLE-ABS-KEY ("lactobacillaceae") OR TITLE-ABS-KEY ("Lacticaseibacillus") OR TITLE-ABS-KEY ("Lactobacillus") OR TITLE-ABS-KEY ("latilactobacillus sakei") OR TITLE-ABS-KEY ("levilactobacillus brevis") OR TITLE-ABS-KEY ("ligilactobacillus salivarius") OR TITLE-ABS-KEY ("Limosilactobacillus") OR TITLE-ABS-KEY ("Pediococcus") OR TITLE-ABS-KEY ("Bifidobacterium") OR TITLE-ABS-KEY ("saccharomyces boulardii") | 194,216 |
| #2 | TITLE-ABS-KEY ("sleep*") | 476,027 |
| #3 | TITLE-ABS-KEY ("placebo*") OR TITLE-ABS-KEY ("clinical trial") OR TITLE-ABS-KEY ("randomized controlled trial") OR TITLE-ABS-KEY (randomized) OR TITLE-ABS-KEY (randomised) OR TITLE-ABS-KEY (randomly) | 3,491,713 |
| #4 | #1 AND #2 AND #3 | 392 |
|  | Limit: March 31, 2022 >> October 1^st^, 2025 | 204 |

**Web of Science**

| No. | Search query | Results |
| --- | --- | --- |
| #1 | AB="probiotic*" OR AB="synbiotic*" OR AB="paraprobiotic*" OR AB="para psychobiotic*" OR AB="psychobiotic*" OR AB="lactobacillaceae" OR AB="Lacticaseibacillus" OR AB="Lactobacillus" OR AB="latilactobacillus sakei" OR AB="levilactobacillus brevis" OR AB="ligilactobacillus salivarius" OR AB="Limosilactobacillus" OR AB="Pediococcus" OR AB="Bifidobacterium" OR AB="saccharomyces boulardii" | 116,251 |
| #2 | AB="sleep*" | 261,195 |
| #3 | AB="placebo*" OR AB="clinical trial" OR AB="randomized controlled trial" OR AB=randomized OR AB=randomised OR AB=randomly | 1,603,431 |
| #4 | #1 AND #2 AND #3 | 151 |
|  | Limit: March 31, 2022 >> October 1^st^, 2025 | 90 |

TOTAL = 388

DUPLICATE = 159

SCREEN = 229
